# Amendment Bridges Habitat-Driven Quality Gaps in *Tetrastigma hemsleyanum* Through Coordinated Regulation of Soil Enzymes and Fungal Communities

**DOI:** 10.3390/plants15060872

**Published:** 2026-03-11

**Authors:** Su’e Zhang, Chaodu Wu, Peikun Jiang, Yinxiu Liu, Chengpeng Huang

**Affiliations:** 1 College of Environmental and Resource Science, Carbon Neutrality Academy, Zhejiang A&F University, Hangzhou 311300, China; 13857049676@163.com (S.Z.); 15870239651@163.com (C.W.); jiangpeikun@zafu.edu.cn (P.J.); 2 Suichang Agricultural and Rural Affairs Bureau, Lishui 323300, China; 3 Zhejiang Provincial Station of Farmland Quality and Fertilizer Management, Hangzhou 310020, China

**Keywords:** *Tetrastigma hemsleyanum*, biochar, flavonoid biosynthesis, soil extracellular enzymes, fungal communities, habitat-specific regulation

## Abstract

*Tetrastigma hemsleyanum* is a valuable medicinal plant whose dryland cultivation typically yields 30–35% lower flavonoid concentration than forest understory systems due to soil and microbial deficiencies. We investigated whether biochar amendment could bridge this quality gap through rhizosphere microecological regulation. Using a split-plot pot experiment with in situ soils from a bamboo forest and a vegetable field, we applied biochar at 2%. Biochar in bamboo forest (MBBC) achieved the highest flavonoid concentrations, exceeding unamended forest and vegetable controls by 22% and 35%, respectively. Biochar effects were habitat-specific. In acidic forest soils (pH 4.95), it raised the pH to 5.61, while in vegetable fields, it boosted leucine aminopeptidase by 159%. Partial least squares path modeling revealed biochar exerted its effects indirectly (indirect effect = 0.88), with soil extracellular enzymes mediating between soil conditions and plant biosynthetic enzymes (PAL, CHS, CHI). Fungal composition was positively associated with biosynthesis (β = 1.68, *p* < 0.01), particularly *Mortierellomycetes*, whereas bacterial diversity unexpectedly exhibited a significant negative correlation with it (β = −0.79, *p* < 0.05). Biochar disrupted *Eurotiomycetes* dominance in forest soils (from 85% to 39%), creating functionally diverse niches that were associated with enhanced flavonoid accumulation. These findings demonstrate biochar functions as an ecological niche regulator, providing a sustainable strategy for high-quality medicinal plant production in non-native habitats.

## 1. Introduction

*Tetrastigma hemsleyanum* Diels et Gilg, a valuable Chinese medicinal plant endemic to southern China, produces flavonoid-rich root tubers with potent anti-inflammatory and antitumor activities [1,2]. In traditional practice, forest understory cultivation yields superior quality compared to dryland greenhouse systems—flavonoid concentration in forest-grown plants typically exceeds dryland cultivation by 30–35% [3,4]. This quality gap poses a critical challenge for scaling up production sustainably. Forest soils provide unique conditions: acidic pH (4.5–5.5), abundant organic matter, and stable microbial communities that dryland agricultural soils lack [5]. While greenhouse shading can replicate forest light conditions, the fundamental soil differences remain unaddressed. Stone-rich forest soils harbor distinct bacterial communities, particularly Bradyrhizobium, that correlate with enhanced tuber growth [5], suggesting soil microecology plays a decisive role in determining crop quality.

Can we bridge this quality gap through soil manipulation? Specifically, can biochar amendment compensate for the soil deficiencies that limit dryland cultivation quality? More fundamentally, what are the soil microecological mechanisms that determine flavonoid biosynthesis across contrasting habitats—physicochemical properties, enzyme activities, or microbial communities? How do these mechanisms differ between forest and agricultural systems? These questions remain unanswered and are critical for developing practical cultivation strategies.

Traditional Chinese medicine recognizes that medicinal plant quality depends on geographic origin—the “Dao-di” concept [6,7]. Modern rhizosphere ecology explains why: Soil microbial communities and extracellular enzyme activities, not just nutrient levels, regulate secondary metabolite biosynthesis in medicinal plants [8,9]. Beneficial microbes enhance flavonoid production through multiple pathways. In intercropping systems, flavonoid secretion from legume roots recruits beneficial rhizobacteria such as *Bradyrhizobium*, establishing mutualistic partnerships that enhance nitrogen fixation [10]. This creates positive feedback loops where flavonoids recruit microbes, which then promote more flavonoid synthesis. *Arbuscular mycorrhizal* fungi mobilize phosphorus and induce systemic resistance [11], while specific rhizobacteria modulate plant hormones and activate defense pathways [12]. Some endophytic fungi can directly produce bioactive compounds identical to their hosts: *Didymella* isolates from *Peucedanum praeruptorum* synthesize coumarins and, when reinoculated, enhance host quality [13]. However, agricultural practices disrupt these partnerships. Continuous monoculture depletes beneficial microbes and accumulates pathogens, causing quality decline in medicinal crops like *Sophora flavescens* [14]. Can we rebuild these soil–plant-microbe partnerships in non-native cultivation systems? Biochar offers a promising approach.

Biochar is produced by pyrolyzing biomass under oxygen-limited conditions. It rehabilitates degraded soils through multiple mechanisms [15]. Its porous structure (300–500 m^2^ g^−1^) provides microbial habitat while improving water and nutrient retention [15]. In acidic soils, biochar’s alkaline ash raises pH and alleviates aluminum toxicity [15]. It also stimulates extracellular enzymes involved in C, N, and P cycling [16].

Critically, bacteria and fungi respond differently to biochar. Bacteria mainly use biochar’s water-soluble components (dissolved organic carbon, mineral nitrogen), while fungi colonize the solid matrix—micropores, recalcitrant carbon, and pH-modified surfaces [17]. This niche partitioning explains their contrasting ecological strategies. Bacterial communities converge toward copiotrophic r-strategists after biochar addition, potentially creating resource competition with plants. Fungal communities, however, show habitat-specific restructuring [17]. Biochar can activate leucine aminopeptidase (LAP) through microbial “nitrogen-mining”—biochar alters nitrogen availability, triggering microbes to produce nitrogen-acquisition enzymes to maintain metabolic balance [18]. Co-application with organic–inorganic fertilizers produces synergistic effects—in acidic orchard soils, combined treatments elevate pH, enhance enzyme activities, and increase bacterial richness more effectively than either amendment alone [19].

In *Tetrastigma hemsleyanum*, phenylalanine ammonia-lyase (PAL), chalcone synthase (CHS), and chalcone isomerase (CHI) regulate flavonoid biosynthesis in response to environmental signals [1,2]. Biochar has been shown to upregulate these biosynthetic enzymes and substantially boost flavonoid concentration [1]. However, existing studies used single habitat types, ignoring how land-use history shapes soil properties and microbial communities.

Based on the above synthesis, we propose the following primary hypothesis: Biochar amendment bridges the habitat-driven flavonoid quality gap in *Tetrastigma hemsleyanum* primarily by restructuring rhizosphere fungal communities and activating soil extracellular enzyme activities, with the magnitude and direction of these effects being habitat-specific rather than universal across soil types. To test this hypothesis, the present study pursues the following three focused research objectives: (1) to quantify whether 2% biochar amendment can compensate for habitat-driven quality differences in *Tetrastigma hemsleyanum* across bamboo forest and vegetable field soil systems; (2) to identify the dominant pathway (soil physicochemical amelioration, enzymatic activation, or microbial community restructuring) through which biochar exerts its effects using PLS-PM; and (3) to evaluate whether bacterial and fungal communities play contrasting roles in mediating plant secondary metabolism, with specific attention to the potential trade-off between bacterial diversity and fungal compositional effects on flavonoid biosynthesis.

## 2. Results

### 2.1. Flavonoid Accumulation and Biosynthetic Enzyme Activities

**Figure 1:**
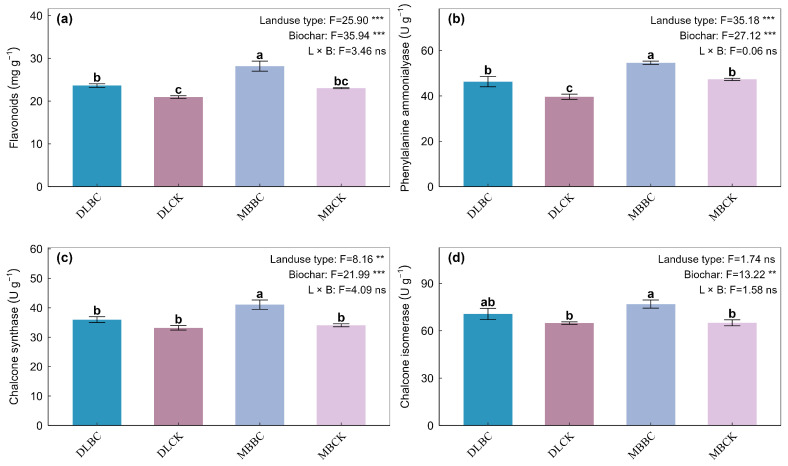
Effects of land-use type and biochar amendment on flavonoid accumulation and key biosynthetic enzyme activities in the root tubers of *Tetrastigma hemsleyanum*. (**a**) Total flavonoid concentration; (**b**) Phenylalanine ammonia-lyase (PAL) activity; (**c**) Chalcone synthase (CHS) activity; (**d**) Chalcone isomerase (CHI) activity. Data are expressed as mean ± standard error (*n* = 6). Different lowercase letters above the bars indicate significant differences among treatments based on Tukey’s HSD test (*p* < 0.05). Abbreviations: DLCK, vegetable field control; DLBC, vegetable field with biochar; MBCK, bamboo forest control; MBBC, bamboo forest with biochar. ** indicates *p* < 0.01; *** indicates *p* < 0.001; ns indicates not significant.

Land-use type and biochar amendment independently influenced total flavonoid concentration and PAL activity in *Tetrastigma hemsleyanum* root tubers (*p* < 0.05), though no interaction between these factors was observed (Figure 1a,b). The MBBC treatment resulted in the highest flavonoid accumulation, achieving increases of 22.26% and 34.59% compared to MBCK and DLCK, respectively. PAL activity peaked under the MBBC treatment, showing a pattern consistent with flavonoid concentration.

Downstream phenylpropanoid enzymes responded similarly. Individual factors influenced CHS and CHI activities (*p* < 0.05), but we detected no interactions (*p* > 0.05). Within the bamboo plantation, biochar increased CHS by 21% and CHI by 18% relative to MBCK (Figure 1c,d).

### 2.2. Soil Properties, Enzyme Activities, and Microbial Diversity

Biochar and land-use type interacted to control soil pH (*p* < 0.001). Biochar had stronger effects in the bamboo plantation than in the vegetable field, raising pH from 4.95 to 5.61 in the former. Land-use type mainly drove SOC and TN (*p* < 0.05). Bamboo plantation soils held higher pools. Biochar tended to increase both, but the interaction was not significant.

**Figure 2:**
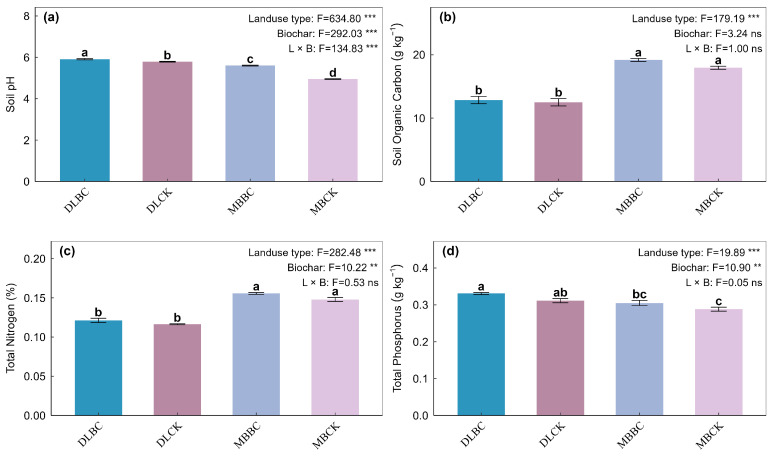
Effects of land-use type and biochar amendment on soil physicochemical properties. (**a**) Soil Organic Carbon (SOC) concentration; (**b**) Soil pH; (**c**) Total Nitrogen (TN) concentration; (**d**) Total Phosphorus (TP) concentration. Data represent the mean of six replicates ± standard error (*n* = 6). Different lowercase letters indicate statistically significant differences between treatments at the *p* < 0.05 level (Tukey’s HSD). ** indicates *p* < 0.01; *** indicates *p* < 0.001; ns indicates not significant.

**Figure 3:**
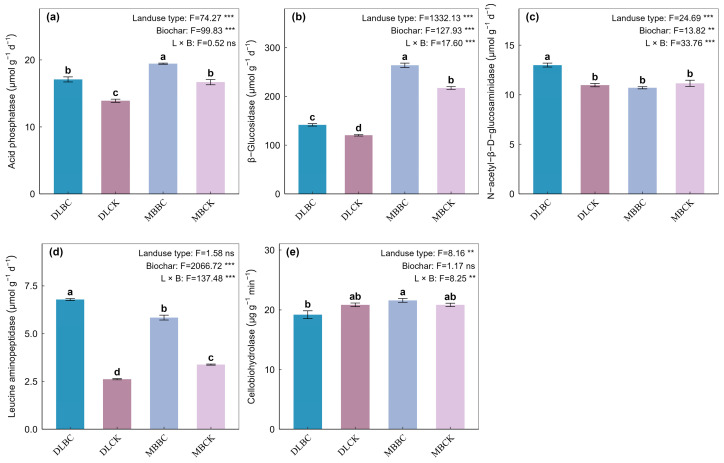
Effects of land-use type and biochar amendment on soil extracellular enzyme activities. (**a**) β-D-cellobiosidase (CBH); (**b**) β-glucosidase (BG); (**c**) Acid phosphatase (AP); (**d**) N-acetyl-β-glucosaminidase (NAG); (**e**) Leucine aminopeptidase (LAP). Data are expressed as mean ± standard error (*n* = 6). Different lowercase letters above the bars indicate significant differences between treatments at the *p* < 0.05 level (Tukey’s HSD). Abbreviations: DLCK, vegetable field control; DLBC, vegetable field with biochar; MBCK, bamboo forest control; MBBC, bamboo forest with biochar. ** indicates *p* < 0.01; *** indicates *p* < 0.001; ns indicates not significant.

**Figure 4:**
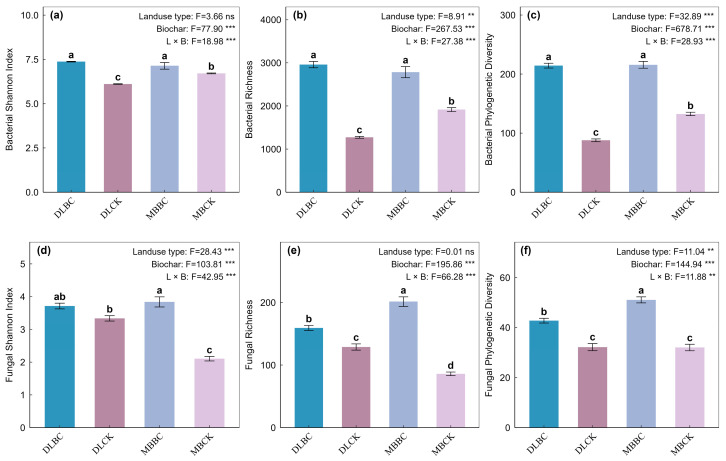
Effects of land-use type and biochar amendment on soil microbial α-diversity indices. (**a**) Bacterial Shannon diversity index; (**b**) Bacterial richness (Observed ASVs); (**c**) Bacterial Faith’s phylogenetic diversity (PD); (**d**) Fungal Shannon diversity index; (**e**) Fungal richness (Observed ASVs); (**f**) Fungal Faith’s phylogenetic diversity (PD). Data are presented as mean ± standard error (*n* = 6). Different lowercase letters above the bars indicate statistically significant differences among treatments based on Tukey’s HSD test (*p* < 0.05). Abbreviations: DLCK, vegetable field control; DLBC, vegetable field with biochar; MBCK, bamboo forest control; MBBC, bamboo forest with biochar. ** indicates *p* < 0.01; *** indicates *p* < 0.001; ns indicates not significant.

Soil enzymes showed specific response patterns. Acid phosphatase responded to land use and biochar independently, without interaction. In contrast, C and N cycling enzymes—β-glucosidase, NAG, LAP, and CBH—showed significant interactive effects (*p* < 0.05). CBH responses were habitat-dependent: Biochar suppressed activity in the vegetable field (from 20.9 to 19.2 μg g^−1^ min^−1^) but stimulated it in the bamboo plantation. LAP responded most strongly, surging 159% in the vegetable field and 73% in the bamboo plantation, Figure 2 and Figure 3. Microbial α-diversity responded significantly to land-use type and biochar amendment (Figure 4). Biochar increased all bacterial indices in both soils. For fungi, biochar boosted Shannon index by 82% in bamboo forest soils versus 11% in vegetable fields, while richness and PD showed similar patterns.

### 2.3. Bacterial and Fungal Community Composition

**Figure 5:**
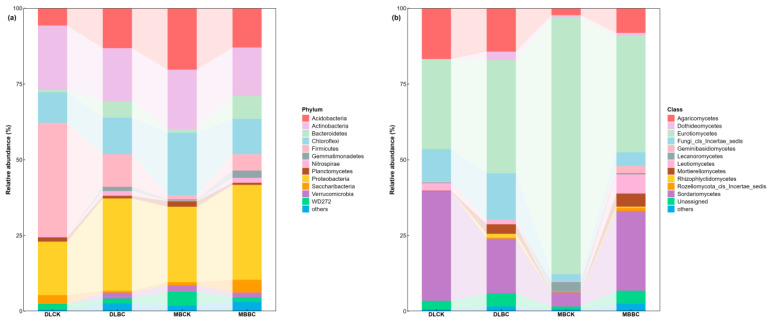
Effects of land-use type and biochar amendment on the relative abundance of soil microbial community composition. (**a**) Bacterial community composition at the phylum level; (**b**) Fungal community composition at the class level. The stacked bars represent the relative abundance (%) of dominant taxa in each treatment. Taxa with relative abundances lower than 1% are grouped as “Others.” Abbreviations: DLCK, vegetable field control; DLBC, vegetable field with biochar; MBCK, bamboo forest control; MBBC, bamboo forest with biochar.

All soils harbored bacterial communities dominated by *Proteobacteria*, *Actinobacteria*, *Firmicutes*, *Acidobacteria*, and *Bacteroidetes* (Figure 5a). Biochar triggered distinct shifts depending on land-use type.

DLCK was dominated by *Firmicutes* (56%). Biochar dramatically restructured this, cutting *Firmicutes* to 19% while boosting *Proteobacteria* (to 34%) and *Actinobacteria* (to 25%)—a shift toward copiotrophic taxa.

In the bamboo forest, *Eurotiomycetes* dominated MBCK with an extreme 85% relative abundance. Biochar selectively suppressed *Acidobacteria* in the bacterial community (from 19% to 10%) and elevated *Bacteroidetes* to 8% in MBBC. For fungi, biochar broke *Eurotiomycetes’ monopoly*, cutting it to 39% while diverse taxa recolonized *Sordariomycetes* (26%), *Agaricomycetes* (8%), *Leotiomycetes* (6%), and *Mortierellomycetes* (4%).

Fungal communities comprised *Eurotiomycetes*, *Sordariomycetes*, *Leotiomycetes*, *Dothideomycetes*, *Mortierellomycetes*, and *Agaricomycetes* (Figure 5b). The vegetable field showed a different pattern. DLCK harbored co-dominant *Sordariomycetes* (36%) and *Eurotiomycetes* (30%). Biochar shifted this balance, reducing *Sordariomycetes* to 18% while promoting *Eurotiomycetes* (37%), *Mortierellomycetes* (3%), and *Dothideomycetes* (3%). Biochar thus acts as an environmental filter, driving divergent fungal succession depending on initial conditions.

### 2.4. Microbial Community β-Diversity

**Figure 6:**
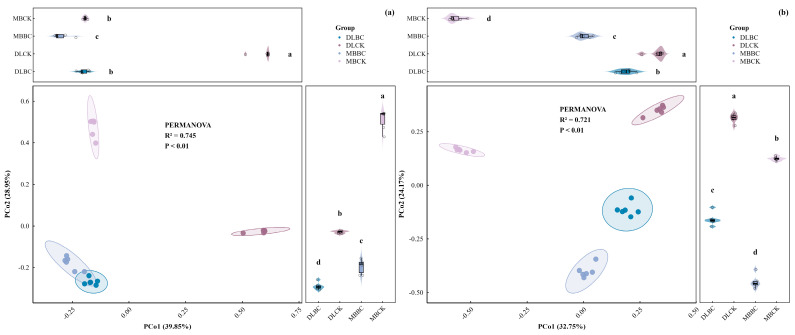
Principal coordinate analysis (PCoA) based on Bray–Curtis dissimilarities, revealing the β-diversity of soil microbial communities under different land-use types and biochar treatments. (**a**) Bacterial community structure; (**b**) Fungal community structure. The percentages on the axes indicate the proportion of total variation explained by the respective principal coordinates (PCoA1 and PCoA2). Distinct clustering indicates significant differences in community composition, as confirmed by PERMANOVA (*p* < 0.01). Data are presented as mean ± standard error (*n* = 6). Different lowercase letters above the bars indicate statistically significant differences among treatments based on Tukey’s HSD test (*p* < 0.05). Ellipses represent 95% confidence intervals for each treatment group. Abbreviations: DLCK, vegetable field control; DLBC, vegetable field with biochar; MBCK, bamboo forest control; MBBC, bamboo forest with biochar.

PERMANOVA confirmed treatments significantly reshaped bacterial (R^2^ = 0.75, *p* < 0.01) and fungal (R^2^ = 0.72, *p* < 0.01) communities, explaining over 70% of compositional variation (Figure 6).

Bacterial PCoA (68.8% variance explained) showed an initial separation according to land-use type. DLCK and MBCK clustered distinctly, whereas biochar triggered convergent migration. Both DLBC and MBBC moved toward the third quadrant, substantially narrowing the gap between them compared to their controls. Biochar-induced microhabitats thus partially overrode land-use history’s divergent effects on bacterial architecture.

Fungal PCoA (56.9% variance) revealed higher heterogeneity and land-use sensitivity. Biochar’s restructuring was most transformative in the plantation. MBCK’s tight initial clustering was dismantled by amendment, causing dramatic displacement toward a novel configuration. Unlike bacterial convergence, biochar drove divergent fungal trajectories across habitats, disrupting existing equilibria and establishing reorganized distributions.

### 2.5. Correlations Among Soil, Microbes, and Plant Metabolism

**Figure 7:**
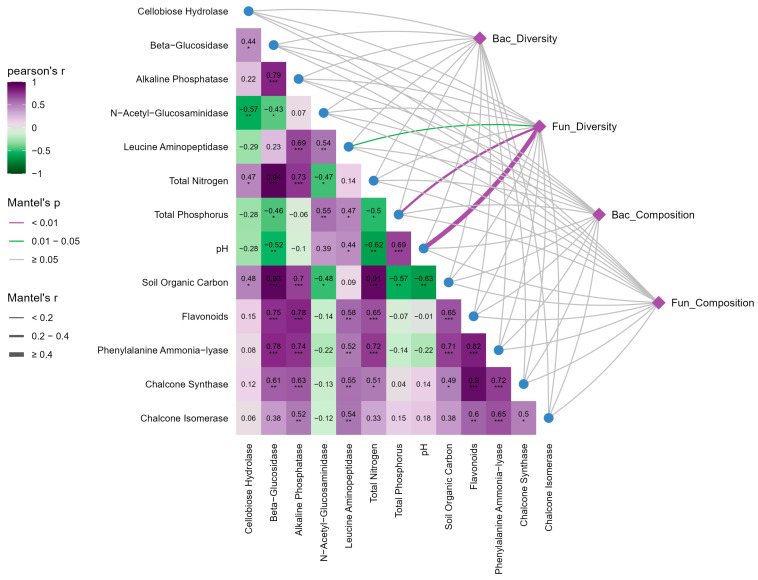
Correlation analysis relating soil biological and physicochemical drivers to *Tetrastigma hemsleyanum* quality. The pairwise comparisons of soil physicochemical properties, soil enzyme activities, and plant quality indices are displayed in the heatmap, with the color gradient representing Pearson’s correlation coefficients (r). Mantel tests were employed to determine the correlations between these variables and four aggregated microbial community attributes: (1) Bac_Diversity (calculated from Bacterial Shannon, Simpson, Richness, and Evenness indices); (2) Fun_Diversity (calculated from Fungal Shannon, Simpson, Richness, and Evenness indices); (3) Bac_Composition (represented by the first two principal coordinates, PCo1 and PCo2, derived from PCoA based on Bray–Curtis distance); and (4) Fun_Composition (represented by fungal PCo1 and PCo2). Edge width corresponds to Mantel’s r statistic, and edge color denotes statistical significance. Abbreviations: Bac_Diversity, Bacterial α-diversity; Fun_Diversity, Fungal α-diversity; Bac_Composition, Bacterial community composition; Fun_Composition, Fungal community composition. Significance levels: * *p* < 0.05, ** *p* < 0.01, *** *p* < 0.001.

Mantel tests showed fungal communities responded more strongly than bacteria to soil properties and plant metabolic factors (Figure 7). For fungal diversity, soil pH was the key driver (r = 0.48, *p* < 0.01). Total phosphorus (r = 0.23, *p* = 0.01) and LAP (r = 0.11, *p* < 0.05) were positively associated with fungal diversity. Fungal composition showed weaker direct links to environmental factors, with only marginal CHI correlation trends.

### 2.6. Land-Use Type-Driven Mechanism: PLS-PM Analysis

**Figure 8:**
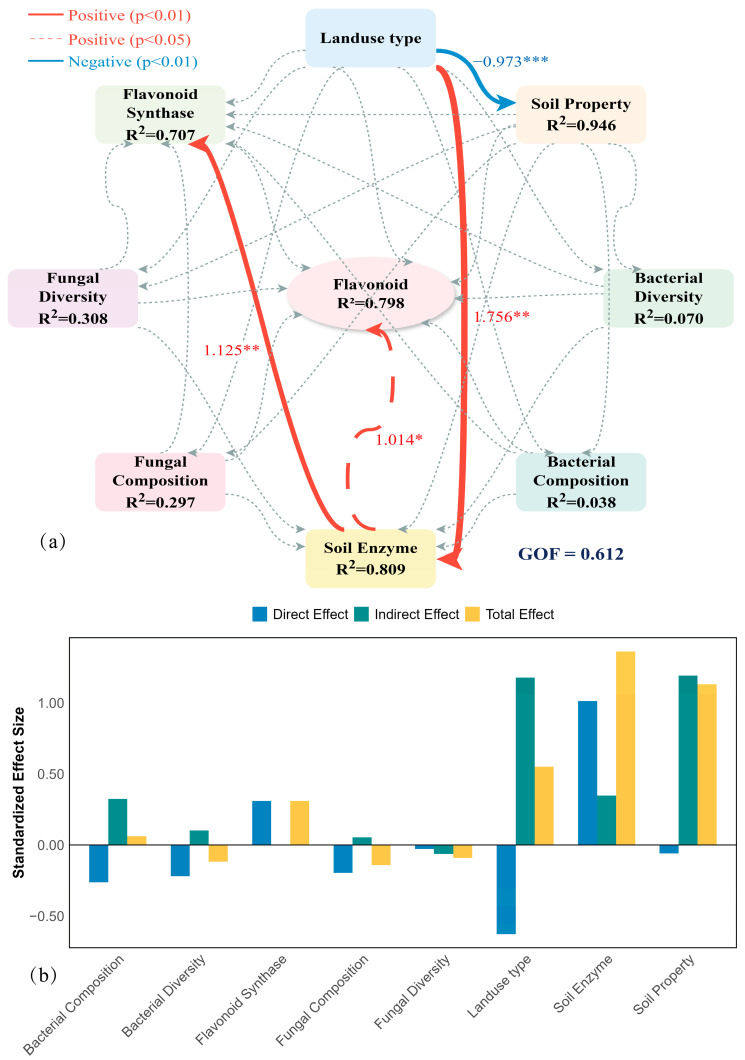
Partial least squares path modeling (PLS-PM) disentangling the land-use type-driven mechanisms of flavonoid quality formation in *Tetrastigma hemsleyanum*. (**a**) Directed path diagram of the structural model illustrating the *statistical pathway model* from land-use type to soil physicochemical properties, microbial community characteristics, soil enzyme activities, plant flavonoid synthase, and flavonoid accumulation. Red and blue arrows indicate *significant positive and negative associations* (*p* < 0.05), respectively, while gray lines denote non-significant pathways (*p* > 0.05). The width of the arrows is proportional to the magnitude of the standardized path coefficients (values shown on arrows). R^2^ values inside the circles indicate the proportion of variance explained for each endogenous latent variable. The model achieves a goodness-of-fit (GOF) of 0.6125. (**b**) Decomposition of standardized direct, indirect, and total effects of biotic and abiotic drivers on total flavonoid accumulation. Latent Variable Construction:Soil Property: Soil organic carbon (SOC), Total nitrogen (TN), Total phosphorus (TP), and pH. Soil Enzyme: β-glucosidase (BG) and Acid phosphatase (AP). Bacterial Diversity: Richness and Evenness indices. Fungal Diversity: Shannon, Simpson, and Evenness indices. Bacterial/Fungal Composition: Represented by the first two principal coordinates (PC1 and PC2) derived from Bray–Curtis distance matrices. Flavonoid Synthase: Phenylalanine ammonia-lyase (PAL) and Chalcone isomerase (CHI). Significance levels: * *p* < 0.05, ** *p* < 0.01, *** *p* < 0.001.

We used PLS-PM to dissect how land-use type, soil properties, microbial communities, and enzyme activities influence flavonoid accumulation. Our optimized model fit well (GOF = 0.61) and explained 80% of flavonoid variation (R^2^ = 0.80), confirming that land-use type and its environmental cascades drive quality differences (Figure 8).

Effect decomposition revealed the total effect of land-use type on flavonoids to be 0.55. Plantation land use showed a direct negative path coefficient (−0.63), but indirect path coefficients (1.18) more than offset this, resulting in a net positive total effect.

Land-use conversion was strongly associated with differences in soil properties (SOC, TN, pH, TP; β = −0.97, *p* < 0.001), explaining 95% of their variation. Although soil properties did not show a significant direct statistical association with flavonoids, their total path effect reached 1.13 through downstream co-variation with biological variables.

Land-use type was strongly associated with soil enzyme activities (BG and AP; β = 1.76, *p* < 0.01). Soil enzymes showed a significant direct positive association with flavonoids (β = 1.01, *p* < 0.05). Effect decomposition confirmed soil enzymes (total effect 1.36) as the model’s most critical mediator. They not only showed a significant positive statistical association with flavonoids directly but also regulated plant biosynthetic enzymes PAL and CHI (β = 1.12, *p* < 0.01), generating substantial indirect contributions.

Bacterial and fungal diversity and composition did not reach significance (*p* > 0.05), though bacterial composition showed a weak positive trend (total effect 0.06), Table 1.

### 2.7. Biochar-Driven Mechanism: PLS-PM Analysis

**Figure 9:**
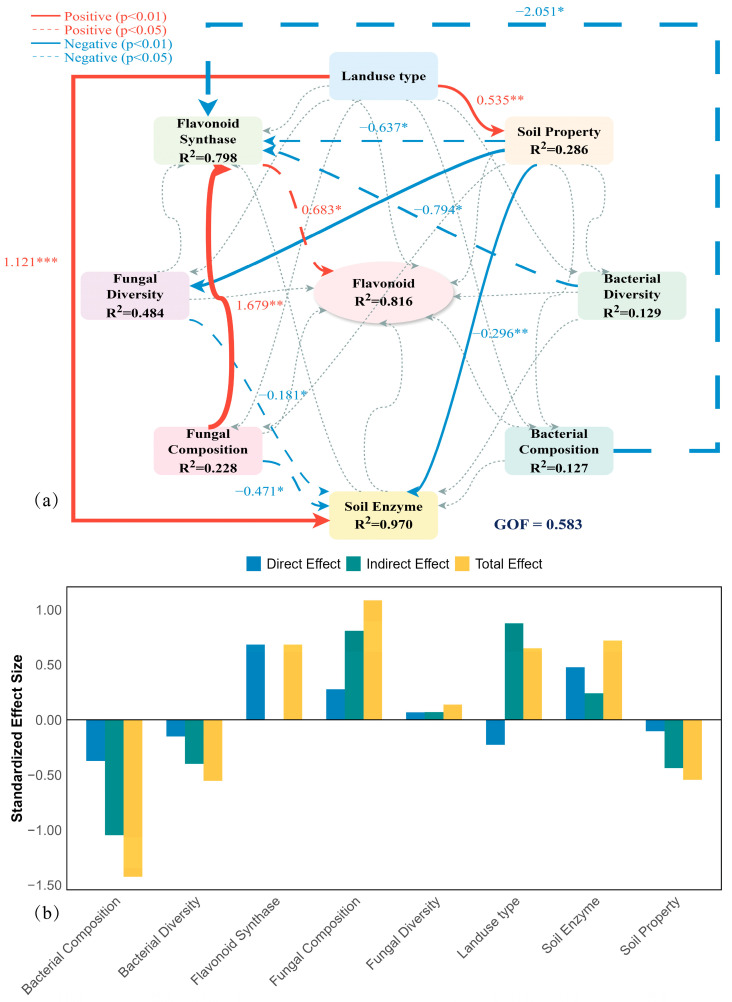
Partial least squares path modeling (PLS-PM) disentangling the biochar-driven mechanisms of flavonoid quality formation in *Tetrastigma hemsleyanum*. (**a**) Directed path diagram of the structural model illustrating the *statistical pathway model* from land-use type to soil physicochemical properties, microbial community characteristics, soil enzyme activities, plant flavonoid synthase, and flavonoid accumulation. Red and blue arrows indicate *significant positive and negative associations* (*p* < 0.05), respectively, while gray lines denote non-significant pathways (*p* > 0.05). The width of the arrows is proportional to the magnitude of the standardized path coefficients (values shown on arrows). R^2^ values inside the circles indicate the proportion of variance explained for each endogenous latent variable. The model achieves a goodness-of-fit (GOF) of 0.583. (**b**) Decomposition of standardized direct, indirect, and total effects of biotic and abiotic drivers on total flavonoid accumulation. Latent Variable Construction: Soil property was constructed from total phosphorus (TP) and pH; soil enzyme from leucine aminopeptidase (LAP) and acid phosphatase (AP); and bacterial diversity from richness and evenness indices. Fungal Diversity: Richness and Evenness indices. Bacterial/Fungal Composition: Represented by principal coordinates (PC1 and PC2) derived from Bray–Curtis distance matrices. Flavonoid Synthase: Phenylalanine ammonia-lyase (PAL), Chalcone synthase (CHS), and Chalcone isomerase (CHI). Significance levels: * *p* < 0.05, ** *p* < 0.01, *** *p* < 0.001.

Our biochar PLS-PM fit well (GOF = 0.58) and explained 82% of flavonoid variation, positioning biochar as a core quality driver (Figure 9). Unlike land-use type, biochar exerted its effects primarily through indirect statistical pathways (total effect = 0.65; indirect effect = 0.88), reshaping the rhizosphere microenvironment rather than directly intervening in metabolism.

Biochar strongly stimulated LAP and AP (β = 1.12, *p* < 0.001) while raising soil pH and available phosphorus (β = 0.54, *p* < 0.01), alleviating original habitat limitations.

Soil enzymes showed positive total effects on flavonoids (0.72), consistent with a biochemical facilitation of quality formation. Fungal composition showed a strong positive association with biosynthetic enzymes PAL, CHS, and CHI (β = 1.68, *p* < 0.01; total effect 1.09). In contrast, bacterial composition (β = −2.05, *p* < 0.05) and diversity (β = −0.79, *p* < 0.05) exhibited significant negative associations with biosynthetic enzymes. Biosynthetic enzymes showed a significant direct positive association with flavonoid concentration (β = 0.68, *p* < 0.05), Table 2.

## 3. Discussion

### 3.1. Biochar Works Differently Between Forest and Agricultural Soils

Soil conditions inherited from different land-uses strongly influenced *Tetrastigma hemsleyanum* quality. Our land-use path model (Figure 8a) showed forest habitat was associated with negative path coefficients on soil properties (β = −0.97). Forest acidity constrains nutrient cycling. Biochar disrupted this.

Biochar raised forest soil pH by 0.65 units, far more than in vegetable fields. This shows biochar works better in low-buffering systems. Continental-scale studies confirm that pH acts as a stronger environmental filter for bacteria than for fungi [20]. This explains why biochar-induced pH shifts triggered more dramatic bacterial community convergence (toward copiotrophic taxa), while fungal responses remained habitat-specific. The alkaline ash (rich in Ca, Mg, K) neutralizes Al^3+^, transforming the legacy effect from suppressive to facilitative. Similar cation additions in tropical forests rapidly improved root growth by relieving multi-nutrient limitations [21]. pH correction unlocked biological responses, not just chemical shifts.

### 3.2. Soil Enzymes Link Biochar to Plant Biosynthesis

Soil extracellular enzymes link soil conditions to plant metabolism. Our biochar model (Figure 9a) showed strong LAP and AP stimulation (β = 1.12, *p* < 0.001).

This supports nitrogen-mining. Song et al. (2020) showed biochar retains nitrogen in micropores, creating localized N-rich patches that trigger microbial enzyme production [18]. Our 159% LAP surge in the vegetable field fits this because agricultural soils have more labile nitrogen than forest soils.

Path analysis revealed that soil enzymes were associated with plant PAL/CHI (β = 1.13) (Figure 8a). This enzymatic cascade may optimize resource allocation between growth and defense. In *Cyclocarya paliurus*, biochar showed a clear growth–defense trade-off: moderate rates maximized total secondary metabolite yield by balancing biomass with per-unit concentration [22]. Our results suggest similar dynamics. Biochar-stimulated soil enzymes may provide sufficient nitrogen for biosynthesis without triggering excessive vegetative growth that would dilute metabolites.

Integrated nutrient management can enhance this effect. Cotton studies show optimized fertilizer regimes improve protein quality by maintaining a steady nutrient supply rather than pulse doses [23]. Similarly, slow-release formulations sustain enzyme activities and crop quality in Chinese chives [24]. These findings support biochar’s slow-release nutrient dynamics as advantageous for medicinal quality formation.

### 3.3. Why Bacteria Inhibit but Fungi Promote Quality

Interestingly, our PLS-PM revealed a negative association between bacterial diversity and flavonoid biosynthesis (β = −0.79, *p* < 0.05). While we currently lack direct empirical evidence, we hypothesize that this pattern may be linked to differences in ecological life-history strategies and transient resource partitioning. Bacteria generally act as fast-growing r-strategists with a high capacity to rapidly assimilate labile carbon (e.g., root exudates) and available nutrients [25]. Consequently, a highly diverse and abundant bacterial community may exert strong exploitative competition in the rhizosphere, rapidly depleting resources that would otherwise be available to plants and beneficial fungi. This intense bacterial competition could restrict niche space for slower-growing fungal taxa (K-strategists) [26], which our data suggest are more directly and positively associated with upregulating flavonoid biosynthetic pathways (PAL, CHS, CHI). Furthermore, excessive bacterial proliferation may alter the internal carbon-nutrient balance of the plant, prioritizing primary metabolism over secondary defense metabolism.

However, we acknowledge that attributing this negative relationship primarily to resource competition remains speculative based solely on amplicon sequencing and correlations. Alternative explanations, such as bacterial-induced microenvironmental shifts or high functional redundancy among bacterial taxa [27], cannot be excluded.

Fungal community composition showed a positive association with quality (β = 1.68). Biochar broke *Eurotiomycetes*’ 85% monopoly in forest soils, opening niches for *Mortierellomycetes*. This fungal-mediated quality enhancement appears general across medicinal plants. In *Panax quinquefolius* (American ginseng), biochar induced microbial and metabolic reprogramming that increased multiple ginsenosides and other bioactive compounds [28]. The mechanism was explicitly microbial: Shifts in rhizosphere and endophytic fungal communities directly correlated with specific secondary metabolite profiles. Co-application of biochar with arbuscular mycorrhizal fungi (AMF) amplified effects—2% biochar plus AMF boosted colonization rates and linked specific microbial taxa to secondary metabolite accumulation [29].

Similar patterns emerge in *Pseudostellaria heterophylla* (Radix Pseudostellariae), where phosphorus-modified biochar at 3–5% increased tuber yield by 69–136% while raising polysaccharides and saponins by 2.9–78.8% [30]. The magnitude (up to 78.8%) exceeds our 22–35% flavonoid increase, suggesting biochar efficacy depends on baseline fertility and metabolite pathway targeted.

Biochar’s labile carbon fractions may play underappreciated roles. Duan et al. (2021) showed biochar incorporation increases dissolved organic carbon and readily oxidizable carbon, directly driving bacterial succession [31]. These active carbon pools likely alter root exudation patterns [12], creating feedback loops between plant metabolism and soil biology. Our finding that LAP activity strongly predicted flavonoid biosynthesis suggests similar root–microbe metabolic coupling in *Tetrastigma hemsleyanum*.

Waqas et al. (2017) combined biochar with an endophytic fungus in soybean, obtaining isoflavone levels above either treatment alone [32]. Biochar provided habitat; fungi secreted hormones and elicitors. Dong et al. (2022) found that legumes secrete flavonoids to recruit *Bradyrhizobium*, which boosts nitrogen fixation [10]. Could *Tetrastigma hemsleyanum* use similar “cry for help” strategies with beneficial fungi? Biochar-supported fungi stimulate flavonoid synthesis, elevated root exudates recruit more fungi, amplifying the cycle.

Specific fungi may be associated with distinct functional outcomes. Mycorrhizal associations are associated with changes in plant physiology and restructure rhizosphere microbiomes [33]. Beneficial bacteria can activate *phoD* genes, mobilizing organic phosphorus [34]. Our high acid phosphatase activities (path coefficient to biosynthetic enzymes = 1.13) suggest P mobilization was critical, possibly mediated by fungal phosphatase production.

### 3.4. Practical Applications

Our results matter for medicinal crop production broadly. Continuous monoculture of medicinal plants like Sophora flavescens depletes beneficial microbes, dropping quality. Biochar can reset degraded soils without chemicals.

The biochar-associated enhancement of secondary metabolism we documented reflects a broader pattern. In Alpinia zerumbet (shell ginger), biochar and biochar–compost combinations increased photosynthesis, reduced oxidative stress, and modulated total phenolic and flavonoid concentration. The biochar–compost blend showed additive effects worth exploring. Under saline stress, 3% biochar to *Satureja khuzistanica* (Khuzestan savory) maintained growth while preserving high essential oil yield and carvacrol concentration [35], demonstrating biochar can sustain secondary metabolite production under abiotic stress. In *Pseudostellaria heterophylla*, elevated CO_2_ combined with biochar produced interactive effects—3% biochar with 1000 ppm CO_2_ maximized tuber yield and polysaccharide concentration, though saponin effects were more nuanced [36]. This suggests biochar applications may need optimization under different atmospheric conditions, relevant for protected cultivation.

Co-application works well. *Pomelo* orchards showed biochar plus organic–inorganic fertilizer beat either amendment alone, elevating pH, enzymes, and bacterial richness [19]. These approaches align with integrated soil fertility management principles proven effective across cropping systems.

For scaling up, use habitat-tailored strategies. Acidic forest soils need pH correction (biochar as lime). Agricultural soils need enzyme activation and microbes (biochar as habitat). This dual role makes biochar versatile for bridging quality gaps.

### 3.5. Limitations and Future Perspectives

While this study elucidates the mechanisms by which land-use legacy and biochar addition drive the quality formation of *Tetrastigma hemsleyanum*, several experimental limitations must be acknowledged. First, although the pot cultivation method employed strictly simulates the actual commercial production practices of *Tetrastigma hemsleyanum*—intentionally designed to prevent competition from dominant native species and ensure tuber harvest—pot systems inherently restrict the unconfined natural architecture and root expansion compared to undisturbed wild growth. Even though the pots were placed in situ within their corresponding habitats (bamboo forest and dryland) to experience natural climatic conditions, including natural rainfall and seasonal temperature fluctuations, the long-term temporal dynamics of soil profile development and carbon cycling may still differ from undisturbed wild conditions. Second, this study utilized only one specific type of biochar (bamboo biochar pyrolyzed at 600 °C). The physicochemical properties of biochar—including pore structure, pH, and labile carbon content—are highly dependent on feedstock materials and pyrolysis temperatures, which subsequently drive divergent microbial responses. Consequently, the findings presented here are specific to this agroecosystem and should not be interpreted as universally applicable to the field production of all medicinal plants. Third, we acknowledge that assigning definitive functional roles to specific microbial groups based solely on 16S rRNA and ITS amplicon sequencing remains speculative, as these techniques primarily resolve taxonomic composition rather than actual functional gene expression. Future studies employing multi-omics approaches—such as metagenomics to ascertain genetic potential and metatranscriptomics to identify active functional genes—coupled with targeted experimental manipulations such as synthetic microbial communities and stable isotope probing, are essential to definitively validate the proposed mechanistic pathways and generalize these findings across diverse soil types and biochar sources.

## 4. Materials and Methods

### 4.1. Study Site and Materials

This study was conducted in 2021 at the Medicine King Valley *Tetrastigma hemsleyanum* cultivation base in Suichang County, Zhejiang Province, China (28.6497° N, 119.1076° E). The region is characterized by a subtropical monsoon climate with a mean annual temperature of 19.2 °C and precipitation of 1560 mm, concentrated between March and September. Annual sunshine duration reaches 1750 h with a 250-day frost-free period, providing suitable conditions for medicinal plant cultivation.

Two contrasting land-use types were selected: bamboo plantation (MB) and vegetable field (DL) soils. The vegetable greenhouse was covered with 70% shade netting to simulate plantation canopy closure.

Healthy *Tetrastigma hemsleyanum* Diels et Gilg seedlings of uniform size (6-month-old, 15–20 cm height) were used as experimental material. Biochar was prepared through slow pyrolysis of bamboo feedstock at 600 °C under oxygen-limited conditions. The biochar contained 440, 9, 13, and 8 g kg^−1^ of organic C, N, H, and S, respectively, with pH 10.37, BET surface area of 387 m^2^ g^−1^, and ash concentration of 5.8%. Major cations included Ca (8.5 g kg^−1^), Mg (3.1 g kg^−1^), and K (22.6 g kg^−1^).

### 4.2. Experimental Design

A split-plot design was employed with land-use type as the main plot factor and biochar application as the subplot factor. Main plots were assigned either bamboo plantation (MB) or vegetable field (DL) soil. Subplots received either no biochar (CK) or 2% biochar (BC). Four treatment combinations were established:

1. DLCK: Vegetable field soil, no biochar;
2. DLBC: Vegetable field soil, 2% biochar;
3. MBCK: Bamboo plantation soil, no biochar;
4. MBBC: Bamboo plantation soil, 2% biochar.

Each treatment included six biological replicates, totaling 24 independent pots.

### 4.3. Cultivation and Management

Custom cylindrical pot devices (diameter 30 cm, height 35 cm, volume ~25 L) were used for container cultivation, with each pot filled with 10 kg of in situ soil from the corresponding habitat. Pots had drainage holes at the bottom to prevent waterlogging.

In December 2021, biochar was thoroughly mixed into DLBC and MBBC soils at 2% (*w*/*w*) and incubated for 3 months to ensure biochar–soil equilibration and initial reactions. In March 2022, three *Tetrastigma hemsleyanum* seedlings were transplanted per pot. Root-zone soil was gently compacted, and establishment irrigation (500 mL per pot) was applied after planting.

In this study, a single biochar application rate of 2% (*w*/*w*) was selected. The rationale for this specific dosage was based on previous empirical studies specifically conducted on Tetrastigma hemsleyanum. Existing literature demonstrates that *Tetrastigma hemsleyanum* exhibits a unimodal dose–response to biochar: A lower dose (approximately 2% *w*/*w*, ~20 mg/g) significantly maximizes fresh root biomass and total flavonoid accumulation, whereas excessive doses (e.g., 4% *w*/*w*) fail to increase root yield due to potential overlocking of essential soil nutrients by biochar’s porous structure [1,37]. Agronomically, a 2% (*w*/*w*) rate corresponds to approximately 20–40 t/ha, widely recognized as an economically feasible rate for sustaining soil health without nutrient immobilization toxicity [38]. We acknowledge that a dose–response design across multiple rates is recommended for future field-scale trials.

Consistent watering and manual weeding were maintained throughout the growing period. Two topdressing applications were performed during the seedling stage—the first one month after transplanting and the second after vine pruning. Each application provided 0.10%/pot of compound fertilizer (N:P_2_O_5_:K_2_O = 15:15:15). Fertigation was employed to distribute nutrients uniformly and prevent seedling burn.

### 4.4. Sample Collection and Preparation

Samples were collected in October 2025 during root tuber harvest (approximately 43 months after transplanting).

Soil sampling: Container devices were removed, and surface litter was cleared. All soil from each pot was collected and thoroughly mixed after removing visible gravel and plant debris. Each soil sample was divided into three portions. The first portion was immediately frozen in liquid nitrogen and stored at −80 °C for DNA extraction. The second portion was stored fresh at 4 °C for enzyme assays. The third portion was air-dried and sieved through a 0.149 mm mesh for physicochemical analysis.

Plant sampling: Complete *Tetrastigma hemsleyanum* root tubers were collected and washed with distilled water. One portion was immediately frozen in liquid nitrogen and stored at −80 °C for enzyme activity assays. Another portion was dried to constant weight at 60 °C, ground into powder, and used for flavonoid quantification.

### 4.5. Analytical Methods

#### 4.5.1. Soil Physicochemical Properties

Soil pH was measured potentiometrically in a 1:2.5 (*w*/*v*) soil:water suspension using a pH meter (FE20, Mettler Toledo, Shanghai, China). Soil organic carbon (SOC) concentration was determined by potassium dichromate oxidation with external heating. Total nitrogen (TN) was determined by Kjeldahl digestion followed by titration. Total phosphorus (TP) was determined by sodium hydroxide fusion followed by molybdenum–antimony colorimetric method.

#### 4.5.2. Soil Extracellular Enzyme Activities

Five enzymes involved in C, N, and P cycling were measured using fluorometric microplate assays: cellobiohydrolase (CBH), β-glucosidase (BG), acid phosphatase (AP), N-acetyl-β-glucosaminidase (NAG), and leucine aminopeptidase (LAP).

Following Bell et al. (2013) [39], 1 g of fresh soil was weighed and mixed with 50 mM acetate buffer to prepare a suspension. Soil suspension, buffer, and 4-methylumbelliferone or 7-amino-4-methylcoumarin-labeled substrates were added to 96-well black plates. After 4 h incubation at 25 °C in darkness, reactions were terminated with 1 M NaOH. Fluorescence was measured using a microplate reader (SpectraMax i3, Molecular Devices, San Jose, CA, USA) at 365 nm excitation and 450 nm emission.

#### 4.5.3. Plant Flavonoids and Biosynthetic Enzymes

Phenylalanine ammonia-lyase (PAL), chalcone synthase (CHS), and chalcone isomerase (CHI) activities were measured using double-antibody sandwich ELISA, following Jiang et al. (2023) [1]. Samples were homogenized in PBS buffer and centrifuged. Supernatant was added to enzyme-coated plates. Absorbance was measured at 450 nm, and enzyme activities were calculated from standard curves.

Dried root tuber powder was extracted with 70% ethanol (1:20 *w*/*v*) using ultrasonic-assisted extraction (40 kHz, 30 min, 60 °C) and centrifuged at 5000× *g* for 10 min. Total flavonoids were quantified using the NaNO_2_-AlCl_3_-NaOH colorimetric method. The extract was mixed with 5% NaNO_2_, then 10% AlCl_3_, followed by 1 M NaOH. Absorbance was measured at 510 nm.

#### 4.5.4. Microbial Community Analysis

Total DNA was extracted using the E.Z.N.A.^®^ Soil DNA Kit (Omega Bio-tek, Norcross, GA, USA). DNA concentration and purity were assessed with a NanoDrop 2000 spectrophotometer (Thermo Fisher Scientific, Waltham, MA, USA).

Sequencing was performed on the Illumina NovaSeq platform (Illumina, San Diego, CA, USA). For bacteria, the V3-V4 region of 16S rRNA was amplified using primers 338F (5′-ACTCCTACGGGAGGCAGCAG-3′) and 806R (5′-GGACTACHVGGGTWTCTAAT-3′). For fungi, ITS1 was amplified using primers ITS1F (5′-CTTGGTCATTTAGAGGAAGTAA-3′) and ITS2R (5′-GCTGCGTTCTTCATCGATGC-3′).

Raw sequencing data underwent quality control using fastp with default parameters. Paired-end reads were assembled using FLASH with a minimum overlap of 10 bp. Chimeric sequences were removed using the UCHIME algorithm. Operational taxonomic units (OTUs) were clustered at 97% similarity threshold using UPARSE. Taxonomic annotation was performed using RDP Classifier with a confidence threshold of 0.7 against the SILVA database for bacteria and the UNITE database for fungi.

### 4.6. Statistical Analysis

Data were organized in Microsoft Excel 2021 (Microsoft Corp., Redmond, WA, USA) and statistically analyzed in SPSS 26.0 (IBM Corp., Armonk, NY, USA). Normality and homogeneity of variance were assessed using Shapiro–Wilk and Levene’s tests, respectively. Data were log_10_ transformed when necessary to meet assumptions of parametric tests.

Two-way analysis of variance (ANOVA) was used to test the effects of land-use type, biochar application, and their interaction on soil physicochemical properties, enzyme activities, and plant parameters. When significant effects were detected, post hoc pairwise comparisons were performed using Tukey’s honestly significant difference (HSD) test with a significance level set at *p* < 0.05.

Microbial community analysis was performed in R. Alpha-diversity indices (Shannon, Chao1, Simpson) were calculated using the vegan package. Beta-diversity was visualized through principal coordinates analysis (PCoA) based on Bray–Curtis dissimilarity matrices. Community structure differences among treatments were tested using permutational multivariate analysis of variance (PERMANOVA) with 9999 permutations in the vegan package. Mantel tests (999 permutations) were employed to assess correlations between environmental factors and microbial community composition. Microbial β-diversity was visualized through PCoA based on Bray–Curtis distances. Community structure differences were tested with PERMANOVA. Mantel tests were employed to correlate environmental factors with microbial communities.

Partial least squares path modeling (PLS-PM) was conducted using plspm in R4.4.2. The model included nine latent variables: land-use type, soil properties, bacterial diversity, fungal diversity, bacterial composition, fungal composition, soil enzymes, plant biosynthetic enzymes, and flavonoid content. Each latent variable was constructed from multiple measured indicators using Mode A.

To ensure statistical clarity and robustness of the PLS-PM, a rigorous model optimization and variable selection process was conducted. Rather than arbitrarily testing multiple alternative structural configurations, the hypothesized structural pathways (inner model) were established a priori based on theoretical ecological cascades (i.e., from soil amendment treatments to soil physicochemical properties, to microbial communities and enzyme activities, and ultimately to plant biosynthetic response and flavonoid accumulation). During model development, two alternative structural configurations were evaluated: one treating bacterial and fungal communities as a unified microbial latent construct, and one separating them into independent latent variables (bacterial diversity, bacterial composition, fungal diversity, fungal composition). The latter model produced substantially higher goodness-of-fit and was therefore retained. This separation was also theoretically motivated by documented divergent responses of bacteria and fungi to biochar amendment [40].

To determine which manifest variables were kept or excluded from latent constructs, model optimization employed a strict two-step filtering process: (1) an initial ANOVA screening removed indicators with non-significant treatment effects (*p* ≥ 0.05) to ensure ecological relevance; (2) a subsequent loading filter removed weak indicators (|loading| < 0.7) to ensure statistical reliability, while maintaining a minimum of two robust indicators per latent variable. Throughout this optimization process, the a priori structural paths remained unchanged. The final model was selected because it maximized the GoF index while preserving full theoretical integrity. Path coefficients were tested by bootstrap resampling (1000 iterations, *p* < 0.05), and effects were decomposed into direct, indirect, and total components.

## 5. Conclusions

Biochar application bridged the quality gap between dryland and plantation cultivation of *Tetrastigma hemsleyanum* by creating a fungal-driven, enzymatically active rhizosphere. Quality formation, followed by biochar, alleviated physicochemical constraints from land-use legacy effects, stimulated extracellular enzymes, and restructured fungal communities, triggering plant biosynthetic pathways. This shows that medicinal plant quality enhancement depends more on functional bio-inorganic habitats than on simple nutrient additions. Targeted rhizosphere engineering centered on enzyme-mediated nutrient cycling and fungal network support thus provides a sustainable strategy for achieving high-quality *Tetrastigma hemsleyanum* production in non-native habitats.

## Figures and Tables

**Table 1:** Standardized loadings of manifest variables for the latent variables in the final optimized PLS-PM assessing the effects of land-use type.

Latent Variable	Manifest Variable	Loading	Significance
Soil Property	Total Nitrogen	−0.92	***
	Soil Organic Carbon	−0.938	***
	Soil pH	0.813	***
	Total Phosphorus	0.75	***
Soil Enzyme Activity	β-Glucosidase	0.96	***
	Acid Phosphatase	0.927	***
Bacterial Diversity	Bacterial Richness	0.991	***
	Bacterial Evenness	0.73	***
Fungal Diversity	Fungal Simpson Index	0.995	***
	Fungal Evenness	0.994	***
	Fungal Shannon Index	0.991	***
Bacterial Composition	PCoA Axis 1	0.898	***
	PCoA Axis 2	0.44	*
Fungal Composition	PCoA Axis 1	−0.988	***
	PCoA Axis 2	0.153	ns
flavonoid synthase	Phenylalanine ammonia-lyase	0.955	***
	Chalcone isomerase	0.849	***
Response	Total Flavonoids	1	***

Note: The significance of the loadings was determined by bootstrapping (5000 resamplings). Asterisks indicate statistical significance: *** *p* < 0.001, * *p* < 0.05, ns *p* > 0.05. Loadings > 0.7 indicate that the manifest variable is a reliable indicator of the latent construct, with the exception of the second principal coordinates (PC2) for bacterial and fungal compositions. Although PC2 loadings were lower, they were retained in the model to capture the multidimensional shifts in microbial community structure not explained by the primary gradient (PC1).

**Table 2:** Standardized loadings of manifest variables for the latent variables in the final optimized PLS-PM assessing the effects of biochar amendment.

Latent Variable	Manifest Variable	Loading	Significance
Soil Property	Soil pH	0.928	***
	Total Phosphorus	0.913	***
Soil Enzyme Activity	Leucine aminopeptidase	0.953	***
	Acid Phosphatase	0.878	***
Bacterial Diversity	Bacterial Richness	0.991	***
	Bacterial Evenness	0.732	***
Fungal Diversity	Fungal Evenness	0.901	***
	Fungal Richness	0.815	***
Bacterial Composition	PCoA Axis 1	0.91	***
	PCoA Axis 2	0.416	*
Fungal Composition	PCoA Axis 2	0.992	***
	PCoA Axis 1	0.122	ns
Flavonoid Synthase	Phenylalanine ammonia-lyase	0.909	***
	Chalcone synthase	0.844	***
	Chalcone isomerase	0.842	***
Response	Total Flavonoids	1	***

Note: The significance of the loadings was determined by bootstrapping (1000 resamplings). Asterisks indicate statistical significance: *** *p* < 0.001, * *p* < 0.05, ns *p* > 0.05. Loadings > 0.7 indicate that the manifest variable is a reliable indicator of the latent construct. Exceptions were observed for specific principal coordinates of microbial composition. For bacteria, PC1 (λ = 0.91) was the primary indicator; however, for fungi, PC2 (λ = 0.99) served as the dominant indicator driving the model, suggesting the variation relevant to quality formation was captured by the second dimension of the fungal community structure.

## Data Availability

The data presented in this study are available on request from the corresponding author.

## Abbreviations

The following abbreviations are used in this manuscript:

AP	Acid phosphatase
BG	β-glucosidase
CBH	Cellobiohydrolase
CHI	Chalcone isomerase
CHS	Chalcone synthase
CK	Control (no biochar)
BC	Biochar treatment
DL	Vegetable field (dryland)
DLCK	Vegetable field control
DLBC	Vegetable field with biochar
GOF	Goodness-of-fit
ITS	Internal transcribed spacer
LAP	Leucine aminopeptidase
MB	Bamboo forest
MBCK	Bamboo forest control
MBBC	Bamboo forest with biochar
NAG	N-acetyl-β-glucosaminidase
OTU	Operational taxonomic unit
PAL	Phenylalanine ammonia-lyase
PCoA	Principal coordinate analysis
PERMANOVA	Permutational multivariate analysis of variance
PLS-PM	Partial least squares path modeling
SOC	Soil organic carbon
TN	Total nitrogen
TP	Total phosphorus
